# A one-transistor organic electrochemical self-sustained oscillator model for neuromorphic networks

**DOI:** 10.1016/j.newton.2025.100207

**Published:** 2025-10-06

**Authors:** Juan Bisquert, Nir Tessler

**Affiliations:** 1 Instituto de Tecnología Química (Universitat Politècnica de València-Consejo Superior de Investigaciones Científicas), Camino de Vera s/n, 46022 València, Spain; 2 Andrew & Erna Viterbi Department of Electrical and Computer Engineering, Technion, Haifa 32000, Israel

**Keywords:** Oscillator neuron, organic electrochemical transistor, neuromorphic computation, Hopf bifurcation

## Abstract

Organic electrochemical transistors (OECTs) operating in wet biological environments offer new possibilities for neuromorphic biosensors and bioelectronics. This work presents a device physics approach to develop an organic spiking neuron using a single OECT combined with passive RC components. The key condition is that charge carrier mobility decreases with ion concentration in the organic conductor. This leads to a Z-shaped current-voltage response that, when coupled with an external load, produces self-sustained oscillations. We model the system as a nonlinear oscillator described by a set of first-order differential equations, exhibiting a stable limit cycle. Through nonlinear dynamics and bifurcation theory, we construct a two-variable fast/slow model and identify the conditions for a Hopf bifurcation that triggers oscillatory behavior. The system’s output can shift between sinusoidal spiking and relaxation oscillations by adjusting the external capacitor. Crucially, this neuron-like behavior is achieved using a single transistor without external amplifiers. This minimalistic design offers a promising pathway toward energy-efficient, low-cost, and biomimetic neuromorphic systems, with strong potential for integration in future bioelectronic devices.

## Highlights

• Spiking achieved using a single organic transistor and passive RC elements
• Negative transconductance enables relaxation oscillations via Hopf bifurcation
• Circuit design is low cost, energy efficient, and mechanically flexible
• Provides guidelines for tuning frequency and achieving stable spiking

## Accessible overview

Organic electrochemical transistors (OECTs) have emerged as promising candidates for building neuromorphic systems—electronic circuits that mimic the behavior of biological neurons—especially in flexible, biocompatible, and low-power applications. While earlier designs typically required multiple transistors or external amplifiers to replicate neural spiking behavior, this theoretical work demonstrates that such complexity can be significantly reduced. By leveraging the intrinsic nonlinear properties of a single OECT and complementing it with simple passive components (resistors and capacitors), we show that self-sustained electrical oscillations—analogous to neural spikes—can be generated. The key is a transistor with a peaked transfer curve, which introduces a region of negative transconductance. In this region, small changes in current lead to counterintuitive changes in voltage, destabilizing the system and allowing oscillations to emerge through a well-defined Hopf bifurcation.

This minimal circuit, inspired by relaxation oscillators, operates without amplifiers or complex feedback networks. It uses the transistor as a fast, active element and an external capacitor as a slow, stabilizing influence. The circuit’s behavior depends sensitively on several tunable parameters (such as applied voltage and resistance), and the analysis offers clear guidelines for achieving the desired dynamic regime. Importantly, the frequency of oscillation can be tuned through external capacitance, enabling alignment with biological timescales (e.g., cardiac or neural rhythms).

The demonstration of robust spiking with a single organic transistor using the classical limit-cycle oscillator model represents a step forward in organic neuromorphic hardware. It highlights how basic physical principles—negative differential resistance, timescale separation, and bifurcation theory—can be harnessed to design energy-efficient circuits with biomimetic behavior. Future work integrating more realistic models and exploring device variability will further clarify the potential of OECT-based neurons for applications in bioelectronics and artificial intelligence.

## Graphical abstract

**Figure:**
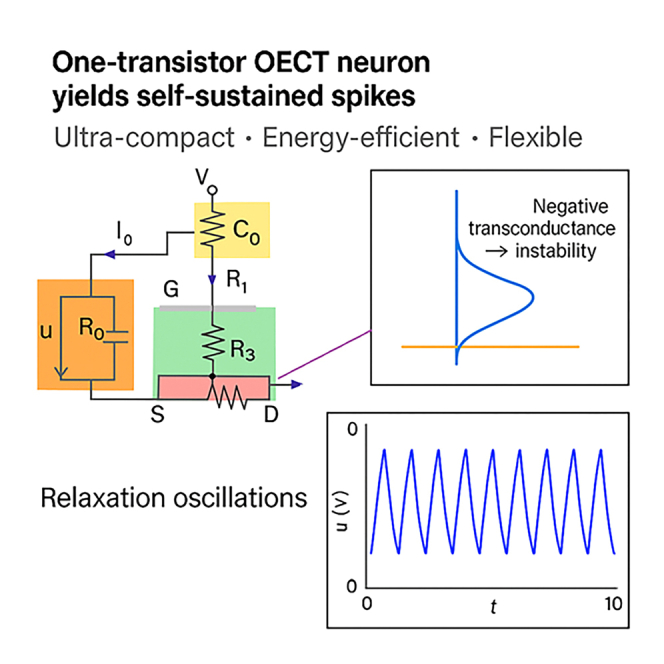

## Teaser

Designs for neuromorphic systems typically involve multiple transistors or external amplifiers. Bisquert and Tessler propose a minimalist neuromorphic circuit using a single organic electrochemical transistor and passive RC elements to generate self-sustained spiking. By exploiting negative transconductance and timescale separation, the system achieves relaxation oscillations without amplifiers. The design offers a compact, energy-efficient, and flexible platform for bioelectronic interfaces and neuromorphic computing, with tunable dynamics and potential for scalable integration.

## Introduction

By mimicking the brain’s structure and dynamics, neuromorphic devices that enable efficient learning and computation emerge as a promising innovative solution to the advances of power-hungry computation and automation technologies.^1^^,^^2^^,^^3^ Unlike traditional computing platforms, these devices operate with exceptional energy efficiency, making them a sustainable alternative. Their capability to replicate the brain’s learning mechanisms allows for continuous adaptation, ideal for real-time decision-making applications.

At the core of this innovation lies the neuron, the fundamental unit for encoding, transmitting, and decoding information in the brain. Natural neurons communicate through spiking signals, which originate from voltage-gated ion channels within the neuron membrane, as described by the Hodgkin-Huxley (HH) model.^4^^,^^5^ This model, formed by a set of nonlinear differential equations, successfully reproduces the characteristic shape and timing of action potentials observed in biological neurons. The model’s influence extends to artificial intelligence, where it informs the development of energy-efficient, spike-based computing systems that mimic the brain’s natural information processing.

The essential feature for encoding information in a neuromorphic circuit is the neuron spiking, in which repetitive signals arise when the neuron fires in response to a constant stimulus. Therefore, a self-sustained oscillator is a fundamental component for replicating the rhythmic and temporal dynamics characteristic of biological neural networks.^6^^,^^7^^,^^8^ These oscillators are nonlinear systems capable of generating continuous, periodic signals without requiring an external periodic input. Instead, they convert a constant, non-periodic source of energy—such as a DC power supply—into sustained oscillations through internal feedback mechanisms.^6^ This ability to autonomously produce rhythmic activity makes self-oscillators essential in neuromorphic systems, where they can emulate the timing and synchronization found in natural neural processes.

In the pursuit of efficient and scalable artificial neural networks (ANNs), implementing neurons using a single transistor presents a compelling advantage over traditional CMOS circuitry. This stems primarily from the need for dynamic, pulse-based operation in several ANN architectures, which significantly reduces energy consumption and enables natural synchronization with other components in complex systems. Conventional CMOS transistors, as typically operated, fall short in reproducing the full spectrum of desired neural and synaptic behaviors under such dynamic conditions. As a result, conventional CMOS-based neuron implementations require multiple interconnected transistors, incurring substantial area and cost penalties. Against this backdrop, the ability to realize a functional neuron using a single transistor not only addresses the limitations of CMOS and emerging technologies but also paves the way for more compact, energy-efficient, and seamlessly integrable neuromorphic systems. Recently, a single silicon transistor neuron was proposed.^9^ The transistor is biased to operate the device on the verge of punch-through conditions while adjusting the resistance of the bulk connection to the ground transistor.

Emerging device technologies based on mixed ionic-electric conductors have the advantage that natural computational mechanisms represented in HH neurons can be reproduced at the materials level. These devices have been explored as alternatives to CMOS due to their compact form factors and potential for mimicking synaptic plasticity. In particular, organic electrochemical transistors (OECTs),^10^^,^^11^^,^^12^^,^^13^^,^^14^^,^^15^ made with organic materials in contact with an electrolyte, are well suited for bioelectronic applications owing to their biocompatibility, biodegradability, and mechanical and electrical matching of biological tissues. The OECT device operation relies on gate voltage-controlled electrochemical ion doping and dedoping of an organic mixed ionic-electronic conductor (OMIEC).^16^^,^^17^^,^^18^ However, in the reported OECT spiking neuron elements,^10^^,^^11^^,^^12^^,^^13^^,^^14^^,^^15^ a combination of several transistors and amplificatory monitoring elements is required.^19^ A single-transistor OECT oscillator has been shown by Fabiano and co-workers, but it relies on strongly asymmetric charge-discharge kinetics.^20^

Self-oscillations^6^ based on the negative differential resistance (NDR) effect have been reported in the last century both in electronic devices, such as Esaki tunnel diodes, Gunn diodes, and resonant tunneling diodes,^21^ and in electrochemical systems.^8^^,^^22^ The standard approach for establishing self-sustained oscillations consists of two elements: a nonlinear system that is destabilized by an intrinsic NDR, such as in binary oxides memristors like VO_2_, combined with a capacitor that produces a rebound effect.^23^^,^^24^ These systems have applications in oscillator-based computation (OBC),^25^^,^^26^ which utilizes a network of oscillators for information processing, with the potential to provide energy-efficient, parallel, and neuromorphic alternatives to conventional computing architectures.

Here we show the method to make a self-oscillating device based on a single OECT following the standard approach, i.e., combining the nonlinear OECT with a support capacitor. We frame the one-transistor OECT circuit as a nonlinear oscillator governed by simple first-order differential equations. Its self-sustained spikes arise from a limit cycle—a stable, closed path in phase space. Using this canonical nonlinear dynamics viewpoint lets us predict when the circuit will oscillate and how its frequency can be tuned.^27^^,^^28^ Previously, we have classified in this way the self-sustained oscillatory bifurcation properties for two-variable S-type current-voltage (I-V) curve systems,^8^ in which the current is a multivalued function of the voltage. This approach aligns with methods used across many scientific fields—such as chemistry, optics, biology, and biochemistry—where systems are naturally described by ordinary differential equations. Both in natural neurons^4^^,^^5^ and in artificial negative resistance-based neuron oscillators,^29^^,^^30^ the repetitive potential spikes are obtained by a Hopf bifurcation that occurs when a parameter value causes a stable equilibrium point to become unstable, leading to the emergence of a stable periodic orbit (a limit cycle).^7^ Limit-cycle oscillators can be characterized by the standard methods of nonlinear dynamics and bifurcation theory.^27^^,^^28^ A key universal insight is that all limit-cycle oscillators, including the OECT-based circuit studied here, exhibit similar dynamical behavior near the onset of oscillations, regardless of the underlying physical mechanisms or scale of the system.

The single-transistor oscillator is not a simple extension of the ordinary oscillatory circuit since the transistor has *three contacts* and requires careful self-actuation by the matched RC elements to regulate the oscillatory conditions. To solve this problem, we extend the early method outlined by Degn^31^ for electrochemical oscillations. Based on a simple dynamical model for the OECT,^32^^,^^33^ we obtain a self-sustained oscillator circuit that is analytically manageable and shows the essential physical insight to establish the conditions of bifurcation and oscillations under a constant applied voltage. The main requirement is a peaked mobility dependence on concentration in the OMIEC, which produces a negative transconductance region that is quite standard in these materials.^34^ The result is a Z-type multivalued current with respect to voltage, which effectively achieves the combination of the standard “negative resistance” feature with a capacitor, enabling oscillations. We show the quantitative conditions for the Hopf bifurcation, which causes a transition from a stable state to an oscillatory mode, according to the variation of parameters.

## Results

### Mechanisms of neuron-like oscillators

The HH model developed by Alan Hodgkin and Andrew Huxley serves as the primary mathematical framework for describing the operation of natural neurons.^4^^,^^35^ When a neuron fires, it generates an action potential that lasts only a few milliseconds and is followed by a refractory period, which prevents immediate reactivation. Natural neurons communicate through spiking signals, which are brief but essential electrical impulses known as action potentials. These signals originate from the activity of voltage-gated ion channels embedded in the neuron’s membrane.

Modern neural networks are constructed using circuits based on CMOS technology, which necessitates complex architectures and a large number of components to simulate spiking computational behavior.^36^^,^^37^ The neurons in these networks rely on relatively large circuits composed of dozens of transistors and sizable integrating capacitors. As a result, replicating the behavior of biological neurons with current design and manufacturing technologies is challenging, leading to high power consumption and poor performance in emulating the human brain.

Another strategy to replicate the bio-neurological phenomena in artificial systems designed for specific sensory-cognitive tasks, recognition, combinatorial problems, and learning is to reproduce computational functionalities using organic materials and device physics. Let us summarize the main required properties.

Neuron-like oscillators operating at a constant input source, which replicate the rhythmic or spiking behavior of biological neurons, can be broadly classified into two categories based on their operating principles: feedback oscillators and negative resistance oscillators.^38^ Each class employs a distinct mechanism to produce sustained oscillatory behavior and offers unique features that can be harnessed in neuromorphic and bio-inspired systems.

Feedback oscillators generate oscillations through the use of a positive feedback loop within an amplification system. This configuration typically involves an active gain element—such as a transistor or operational amplifier—combined with a frequency-selective feedback network. Oscillation in such systems is sustained by satisfying the Barkhausen criteria, which require the loop gain to be unity and the total phase shift around the loop to be zero or an integer multiple of 360°.^39^ In neuron-like systems, feedback oscillators often exhibit nonlinear dynamics that result in periodic spiking or bursting behavior. Feedback oscillators have been formed with OCETs.^10^^,^^19^

In contrast to feedback oscillators, negative resistance oscillators rely on the presence of a component that exhibits an NDR, wherein an increase in voltage results in a decrease in current within a certain operating range. This property allows the oscillator to compensate for energy losses in a resonant circuit, thereby sustaining oscillations. Another central component is the “tank circuit” that contains opposing phase elements, i.e., a capacitor and an inductor, which provides a resonant condition.

In the context of neuromorphic behavior, negative resistance devices are particularly valuable for their ability to replicate the excitable, threshold-based responses seen in real neurons. The vanadium dioxide (VO_2_)-based oscillator, which exploits the insulator-to-metal phase transition (IMT), is widely employed in computation with coupled oscillators.^25^^,^^26^ Oscillators using several OECTS that provide the negative resistance mechanism have been reported.^15^

**Figure 1:**
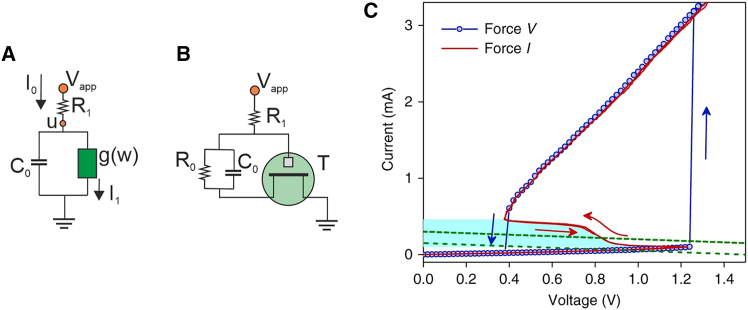
Oscillatory circuits and vanadium dioxide example (A) Standard oscillatory circuit with a two-terminal nonlinear element (green) with conductance function $g\left(w\right)$, where $w=I_{1}$. (B) The single-OECT oscillator circuit suggested in this paper. (C) Two-terminal quasi-d.c. voltage-controlled (force V) and current-controlled (force I) I-V characteristics of a VO_2_ active memristor device and (B) an oscillatory circuit. A wide hysteresis loop exists in the voltage-controlled mode due to the Mott transitions (blue arrows). The same Mott transitions are manifested by an S-shaped NDR regime (highlighted by cyan) with a much narrower hysteresis (red arrows) in the current-controlled mode. In its resting state, the resistor load line intersects with its I-V loci outside the NDR regime (green dotted line). An input current or voltage stimulus can shift the load line into the NDR regime (green dashed line) and elicit an action potential generation (spiking). Reproduced from Yi et al.,^29^ licensed under a Creative Commons Attribution (CC BY 4.0) license.

Negative resistance oscillators are based on a device with negative resistance combined with auxiliary RC elements, as shown in Figure 1A. The method is quite general and can be applied to different material devices.^40^ Negative resistance oscillators operate by the occurrence of a Hopf bifurcation, which requires at least two variables, a fast destabilizing variable and a slow stabilizing variable, usually called a slow-fast dynamical system.^41^ The heart of the Hopf bifurcation property is an S-shape of the I-V curve as shown in Figure 1C, including a negative resistance sector in the transition region (red line, measured galvanostatically), situated between the bistable low- and high-conductance lines (blue points, obtained potentiostatically), associated with the IMT caused by temperature-dependent transport effects.

In the context of nonlinear dynamics and bifurcation theory, an oscillator is well described by a number of first-order differential equations with certain special properties. We show a specific example for Figures 1A and 1C in the methods section. The bifurcation properties of S-oscillators have been amply described.^42^^,^^43^ A progressive passage occurs from harmonic oscillations near the bifurcation point to pulsed, triangular relaxation oscillations.^44^

Many works on OECT neurons have focused on building biology-like spiking features by reproducing the whole HH circuit.^10^^,^^13^^,^^19^ However, such mathematical complexity is not always necessary. There is a long tradition of simplified models with only two differential equations that provide realistic spiking features.^45^ These are more manageable than HH for the design of large networks.

Here, we aim to design a simple, two-contact, physically plausible two-equation oscillator model, based on a *single* OECT, that does *not* contain a voltage comparator. The circuit, to be discussed below, is shown in Figure 1B. This single-OECT neuron operates without supporting amplifiers and provides a route with minimal internal circuitry to replicate the functional operations of the neuron for the formation of perception-responsive circuits. It can operate in an integration function in ordinary ANNs,^46^ and it can also be applied in OBC networks.^25^^,^^26^ Furthermore, the oscillatory properties, such as oscillatory frequency and onset voltage, can be controlled by the external RC elements to modulate the network requirements.

The main condition is a negative transconductance sector^10^^,^^17^ in the OECT. In contrast to the simplicity shown in Figures 1A and 1C, based on a two-contact memristor device, the Z-type I-V curve construction for Figure 1B is more challenging in the case of the OECT, as the auxiliary RC elements are connected between source and gate contacts (see below). To make the oscillator, we need to convert the three-terminal transistor into a spiking two-terminal device.^31^ We will describe several examples and criteria to identify the oscillatory domains.

The next preliminary step is a description of the features of the OECT.

### OECT model

**Figure 2:**
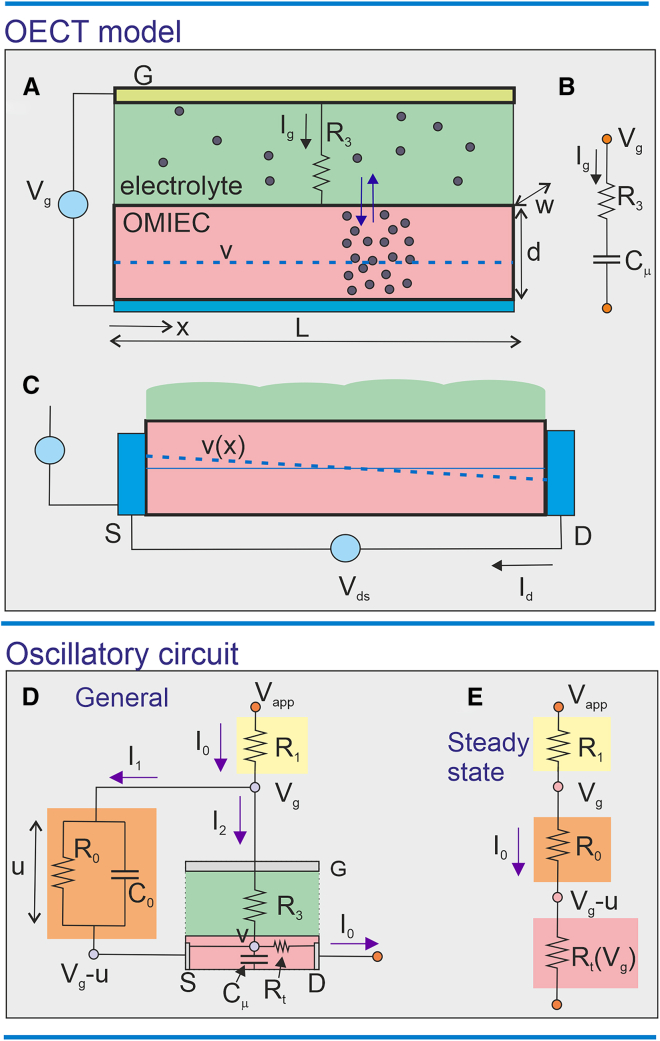
Schemes of the OECT and oscillatory circuit (A–C) Scheme of the OECT. The green zone is the electrolyte, yellow the gate contact, and pink the channel. (A) The measurement of the ion insertion current between gate electrode and the substrate of the film (blue). (B) The equivalent circuit corresponding to (A). (C) The measurement of the drain-source current, with a tilted v(x). (D and E) Circuit model showing node voltages and branch currents. The green-pink zone is the OECT, with gate (G), source (S), and drain (D) contacts indicated. The yellow and orange zones are externally connected elements. (D) General circuit. (E) DC stationary condition, $V_{s}=V_{g}-u$, in which all capacitive currents are 0.

To build a limit-cycle oscillator with matched resistance, we need to formulate the dynamic model of the OECT, which will be combined with support RC elements. Time-dependent models of OECTs can be quite involved, and here, we aim to develop a simple model that is sufficient to show the properties of bifurcation and oscillations, as outlined in Figures 2A–2C. The main physical feature of the OECT is that the OMIEC film exchanges ions with the electrolyte, according to the applied gate voltage $V_{g}$ and the internal electrochemical potential v.^32^^,^^33^ This v will be our internal fast variable corresponding to w in Equation 44 of the methods section. By charge neutrality, the potential sets the charge density and the electronic conductance.

#### Charging the OMIEC film

Let us describe the quantitative model of the OECT in Figures 2A–2C. The number density of ions in the OMIEC layer is a(v). The total electrical charge is

(Equation 1) Q=qLdwa(v).

Here, q is the elementary charge; L,d,andw are the dimensions of the film; and 0≤x≤L is the position along the channel as indicated in Figure 2A. If we apply a small step of the gate voltage ${\Delta V}_{g}$, there will be a step of charge inserted or extracted:

(Equation 2) $$\Delta Q=qL\;w\;d\frac{da}{dv}\Delta V_{g}\text{.}$$

The chemical capacitance $C_{\mu }$ is the derivative of the concentration of a charge carrier with respect to the electrochemical potential.^47^^,^^48^

(Equation 3) $$C_{\mu }=qLwd\;\frac{da_{eq}}{dv}$$

The general concept of a chemical capacitance appears in the transmission line representation of the impedance of mixed ionic-electronic conductors and in the analysis of electrochemical solar cells. In general, one can obtain the chemical (or electrochemical) capacitance by several ordinary electrochemical techniques^49^^,^^50^^,^^51^: electrochemical impedance spectroscopy (EIS), cyclic voltammetry (CV) with step integration, electrochemical quartz crystal microbalance (EQCM), spectroelectrochemistry, chronopotentiometry, chronoamperometry, the potentiostatic intermittent titration technique (PITT), and the galvanostatic intermittent titration technique (GITT). In the OMIEC, the chemical capacitance is an insertion capacitance, which makes OMIECs suitable for use as a battery or capacitor.^52^^,^^53^^,^^54^ Indeed, integrating OMIECs into cathode formulations has led to improved ionic and electronic conductivity, resulting in enhanced discharge capacities and cycling stability.^55^

The chemical capacitance in Equation 3 is proportional to the volume of material, and it is often denominated “volume capacitance” in the literature of OECTs. The equivalent circuit for Figure 2A, including the chemical capacitance, is shown in Figure 2B. For small-signal AC conditions, an equivalent circuit of the transistor can be established as discussed in Bisquert et al.^32^ and Bisquert and Keene.^33^

In many cases, e.g., in PEDOT:PSS films,^33^^,^^48^ the OMIEC is characterized by a constant chemical (or volume) capacitance $C_{\mu }$ with respect to the internal voltage v, which means a linear dependence on charge with respect to voltage, as shown in examples from the literature in Figure S1. Here, we assume a voltage dependence of the form

(Equation 4) $$a\left(v\right)=\gamma_{0}\left(v_{A}+v\right)\text{.}$$

The $\gamma_{0}$ is the charging rate and $v_{A}$ is a constant that establishes the background carrier density ($p_{0}=\gamma_{0}v_{A}$) when v=0. We obtain

Equation 5) $$C_{\mu }=qLwd\;\gamma_{0}\text{.}$$

In the equilibrium situation of Figure 2A, the gate current is $I_{g}=0$. We have a homogeneous charging at the imposed gate potential, $v=V_{g}$.

(Equation 6) $$Q=qL\;d\;w\;a\left(V_{g}\right)$$

Under transient operation $V_{g}\left(t\right)$, the gate current is not zero, as it is charging the chemical capacitance, and we have^32^^,^^33^

(Equation 7) $$I_{g}=\frac{dQ}{dt}=C_{\mu }\frac{dv}{dt}\text{.}$$

Then, v differs from $V_{g}$ due to the ohmic drop at the electrolyte resistance $R_{3}$ (or by the film diffusion resistance in other cases^32^). The relation of potentials is described by the expression

(Equation 8) $$V_{g}=R_{3}I_{g}+v\text{.}$$

Therefore, combining Equations 7 and 8, the transient behavior of the potential/charge in the OECT is governed by the equation^32^^,^^33^ that corresponds to Figure 2B:

(Equation 9) $$C_{\mu }\frac{dv}{dt}=\frac{1}{R_{3}}\left(V_{g}-v\right)\text{.}$$

The time constant for charging the OECT is $\tau_{\mu }=R_{3}C_{\mu }$. A more general model of the transient dynamics is described in Bisquert et al.^32^

We remark that a constant chemical capacitance is chosen for simplicity, but it is not a necessary restriction of our model. One can obtain the chemical capacitance by the indicated electrochemical techniques.^49^^,^^50^ Based on the function determined, one has a voltage-dependent $C_{\mu }\left(v\right)$ in Equation 7.

#### The drain current

The drain current measured in the transistor configuration of Figure 2C is

(Equation 10) $$I_{d}=-\frac{q}{L}d\;w\;\mu \left(a\right)\;a\left(v\right)\;V_{ds}\text{,}$$

where μ is the mobility and $V_{ds}$ the drain-source voltage, which, according to Figure 2B, is defined as $V_{ds}=v\left(L\right)-v\left(0\right)$. Then, we have

(Equation 11) $$I_{d}=-\frac{1}{R_{t}\left(v\right)}\;V_{ds}$$

in terms of a transversal resistance $R_{t}$^33^:

(Equation 12) $$R_{t}\left(v\right)=\frac{L}{q\;d\;w\;\mu \left(a\right)a\left(v\right)}\text{.}$$

In essence, Equations 9 and 11 form the standard Bernard-Malliaras model for transistor transients.^56^ Following this approach, a term $\left(f_{B}\;C_{\mu }\;dv/dt\right)$ for the transient charging can be added in Equation 11, with a constant $f_{B}\approx 0.5$, but then the analysis of bifurcations becomes significantly more complicated.

The density-dependent mobility μ(a) can be expressed as a function of the voltage, using a constant $\mu_{0}$ and a function M(v), as follows:

(Equation 13) $$\mu \left(v\right)=\mu_{0}\;M\left(v\right)\text{.}$$

Combining Equations 4 and 13, we can write

(Equation 14) $$I_{d}=\frac{1}{r_{A}}\;M\left(v\right)\;\left(v_{A}+v\right)V_{ds}\text{,}$$

where we have introduced the constant resistance

(Equation 15) $$r_{A}=\frac{L}{\;q\;d\;w\;\mu_{0}\;\gamma_{0}}\text{.}$$

The transversal resistance is

(Equation 16) $$R_{t}\left(v\right)=\frac{r_{A}}{M\left(v\right)\left[v_{A}+v\right]}\text{.}$$

We remark that the capacitance in Equation 5 is obtained for homogeneous charging, while in the operation mode, there is a tilt of the internal voltage v along the channel, shown in Figure 2C. Both features are compatible if the gradient is small, which requires a high-mobility organic conductor.

#### The negative transconductance

Antiambipolarity producing an inverted V-shape in the drain current (Figures S2A and S2B)^10^ is typically achieved through a partially stacked pn-heterointerface in the transistor channel, which is often composed of 2D atomically thin films or an organic semiconductor.^57^ Another mechanism for decreasing the conductance is a finite density of states.^58^ The conductance in organic films is often carried by polarons that hop between localized sites. At high concentrations, the density of states can be considerably filled, the available sites for hopping decrease, and the mobility decreases (see Figure S2C).^34^^,^^47^ Consequently, the drain current decreases at the increased gate voltages that produce densities exceeding 50%, and the transconductance becomes negative, as reported in some cases.^10^^,^^34^ This property, which is essential for oscillatory behavior, has been reported in other transistor technologies as well.^59^

### Oscillatory circuit model

The oscillatory circuit structure is shown in Figure 2D, and a simplified scheme is shown in Figure 1B. It consists of the transistor described in Figure 2C and three external elements: a parallel $R_{0}C_{0}$ circuit (orange) and a series resistance $R_{1}$ (yellow). $V_{app}$ is the applied voltage, $V_{g}$ is the gate voltage, and u is the internal voltage in the parallel $R_{0}C_{0}$ circuit. The external elements are described by Kirchhoff’s rules:

(Equation 17) $$V_{g}=V_{app}-I_{0}{\;R}_{1}\text{,}$$

(Equation 18) $$I_{1}=C_{0}\frac{du}{dt}+\frac{u}{R_{0}}$$

(Equation 19) $$I_{0}=I_{1}+I_{2}\text{.}$$

#### Simple analytical model

The set of model equations (Equations 9, 16, 17, 18, and 19) is complete. Adopting Equation 4 for a(v), to form a specific transistor model, we need to specify the property $R_{t}\left(v\right)$, which establishes the transport characteristics. We are interested in producing an analytically simple illustration case that shows the dynamical structure of the oscillatory system. Therefore, we introduce some specific modeling assumptions. The following calculations do not have a direct connection with normal OECT properties. Another model with realistic OECT parameters will be developed later on.

To enable the oscillations, a specific mobility with the decreasing property of Figure S2 is needed. For the sake of simplicity, we choose a polynomial fraction of the type

(Equation 20) $$M\left(v\right)=\frac{1}{1+v^{3}}$$

(see Figure S3A). Let us define the auxiliary function

(Equation 21) $$r_{d}\left(v\right)=\frac{r_{A}}{M\left(v\right)}\text{,}$$

and we have

(Equation 22) $$r_{d}\left(v\right)=r_{A}\left(1+v^{3}\right)\text{.}$$

The transversal resistance is

(Equation 23) $$R_{t}\left(v\right)=\frac{r_{d}\left(v\right)}{v_{A}+v}\text{.}$$

#### Stationary operation

In stationary conditions (Figure 2E), we have $I_{2}=I_{g}=0$, and the channel potential is fixed to the gate voltage: $v=V_{g}$. Moreover,

(Equation 24) $$u=V_{g}-R_{t}I_{0}$$

(Equation 25) $$V_{g}=V_{app}-R_{1}I_{0}\text{.}$$

The transfer curve is

(Equation 26) $$I_{d}=-\frac{v_{A}+V_{g}}{r_{A}\left(1+V_{g}^{3}\right)}\;V_{ds}$$

It is shown in Figure S3B.

To obtain the oscillation domain, it is convenient to express the stationary curve in two different dependencies of the total current $I_{0}$. First, using

(Equation 27) $$u=V_{app}-\left(R_{1}+R_{t}\right)I_{0}$$

and Equation 24, we can obtain the dependence $u\left(I_{0},V_{app}\right)$:

(Equation 28) $$u\left(I_{0}\right)=V_{app}-\left[R_{1}+\frac{r_{d}\left(V_{app}-R_{1}I_{0}\right)}{v_{A}+V_{app}-R_{1}\;I_{0}}\right]I_{0}\text{.}$$

From Equation 25, we have

(Equation 29) $$u\left(I_{0}\right)=V_{app}-\left[R_{1}+r_{A}\frac{1+{\left(V_{app}-R_{1}\;I_{0}\right)}^{3}}{v_{A}+V_{app}-R_{1}\;I_{0}}\;\right]I_{0}\text{.}$$

**Figure 3:**
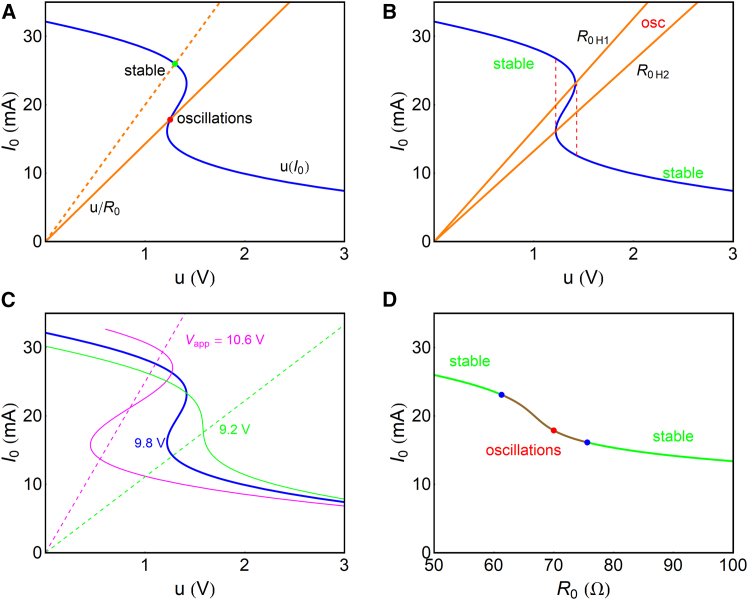
Transconductance curves and oscillatory regimes (A) The stationary current $I_{0}$ obtained from $u\left(I_{0}\right)$ (blue) and from $u/R_{0}$ (orange). $V_{app}=9.8\;V$, $R_{0}=70\;\Omega$ (orange line), and $R_{0}=50\;\Omega$ (dashed orange line) $\mu \left(v\right)=\mu_{0}/\left(1+v^{3}\right)$. The intercept indicated by the red point is the stationary value that leads to oscillations, and the green point gives a stable current-voltage point. (B) $u\left(I_{0}\right)$ curve and the orange lines of the Hopf bifurcation resistances $R_{0H1}=61.20\;\Omega$ and $R_{0H2}=75.59\;\Omega$ for $V_{app}=9.8\;V$. These lines separate the stable and oscillatory regions of the blue curve. The red dashed lines indicate the bistable region of the blue curve. (C) Shape of the $u\left(I_{0}\right)$ curve at different values of $V_{app}$. At 10.6V (pink), the intercept of $u/R_{0}$ produces two stable points. At 9.2V (green), there is no Z-shape. (D) Current as a function of $R_{0}$ for $V_{app}=9.8\;V$, indicating the points (blue) of the Hopf bifurcation, the stable regions (green), and the oscillatory region (brown) showing the red point of (A). Parameter values: $R_{1}=300\;\Omega ,r_{A}=15\;\Omega V,\text{and}\;v_{A}=3\;V\text{.}$

This curve, shown as the blue line of Figure 3A, gives the variation of u when the current $I_{0}$ is changed, corresponding to different values of $R_{0}$.

Secondly, from the orange zone of Figure 2D, we obtain the stationary curve $I_{1}=u/R_{0}$, corresponding to a load line. This is indicated in the orange lines of Figure 3A. When the orange load line $u/R_{0}$ intercepts the blue curve, a stationary point of the whole circuit is obtained that corresponds to the expression

$$V_{app}=\left(R_{0}+R_{1}+R_{t}\right)I_{0}$$

(Equation 30) $$=\left[R_{0}+R_{1}+\frac{r_{d}\left(V_{app}-R_{1}I_{0}\right)}{v_{A}+V_{app}-R_{1}\;I_{0}}\right]I_{0}\text{.}$$

Two examples are indicated in the red and green points in Figure 3A. The variation of $R_{0}$ moves the intercept and changes the steady-state current, as shown in Figure 3D.

We remark that the blue line in Figure 3A has a Z-shape, which contains a bistable region of upper and lower branches and an unstable middle branch, as indicated in Figure 3B in the red dashed lines.

In the analysis of a system’s stability, it is well known^60^ that oscillation in Z- and S-shaped oscillators at fixed voltage $V_{app}$ will occur when the load orange line intersects the unstable zone of the blue line, where $du/dI_{0}>0$, as shown in Figure 3A as the red point. There are two extreme resistance values, $R_{0H1}\;\text{and}\;R_{0H2}$, that meet this condition; the fold points are shown in Figure 3B. These values correspond to the Hopf bifurcation that is described below. A resistance out of this interval intersects with the curve at $du/dI_{0}>0$ and produces a stable point, shown as the green point in Figure 3A.

Note that the change of applied voltage $V_{app}$ modifies the curve of Equation 29, as shown in Figure 3C. In the pink curve, there are three intersection points, of which two are stable. In the green curve, the intercept is at $du/dI_{0}<0$. Neither situation can produce limit-cycle oscillations.

### Dynamical system and bifurcation

We now formulate the dynamical system for the time variation of the above set of equations, as in Equations 43 and 44. This is a standard approach to establish the oscillatory and linear stability properties for S-type oscillators.^8^

At fixed $V_{app}$, the system contains two variable voltages (u,v). The differential Equations 9 and 18 can be expressed in terms of functions F,G (see the methods) as follows:

(Equation 31) $$\frac{du}{dt}=F=\frac{1}{C_{0}}\left(-\frac{u}{R_{0}}+\frac{V_{app}-u}{R_{1}+R_{t}}-C_{\mu }\;G\right)$$

(Equation 32) $$\frac{dv}{dt}=G=\frac{1}{R_{3}C_{\mu }}\left(-v+\frac{R_{1}}{R_{1}+R_{t}}u+\frac{R_{t}}{R_{1}+R_{t}}V_{app}\right)\text{.}$$

Note that these equations are fully general for any model described by Figure 2, according to the specific $R_{t}\left(v\right)$.

**Figure 4:**
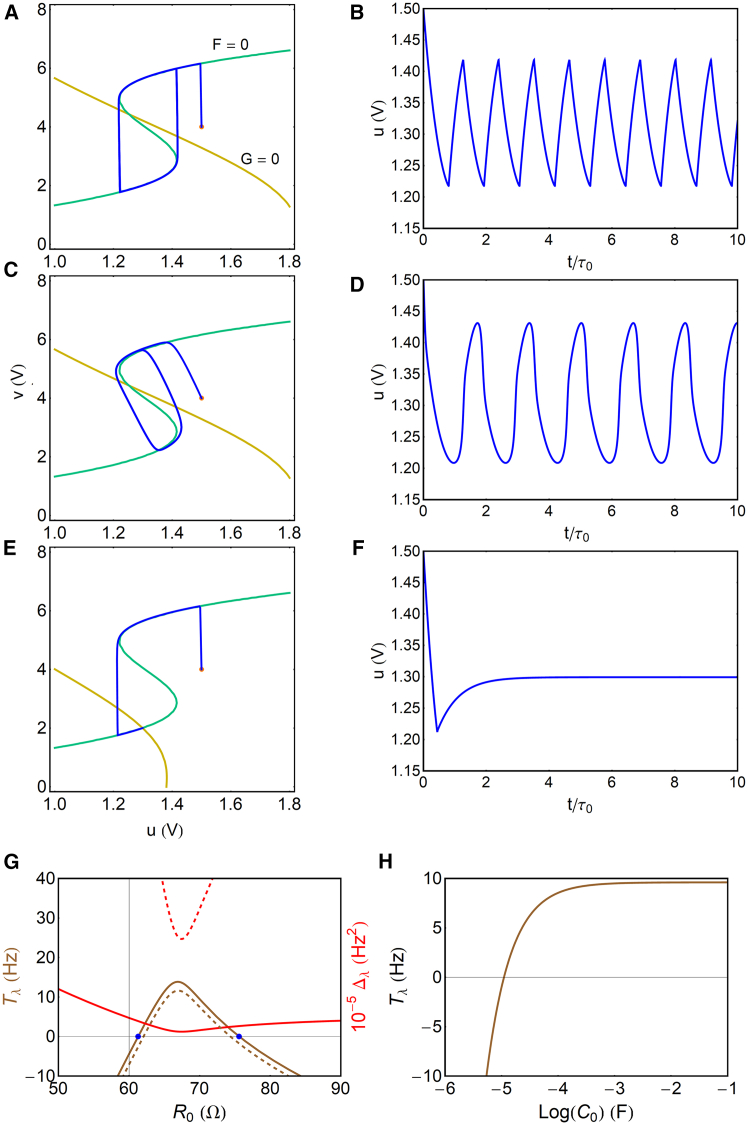
Oscillatory properties of the model (A–F) Phase portrait plot, nullclines (A, C, and E), and trajectory of u voltage (B, D, and F) in the dynamical evolution. The orange point is the initial condition. Parameters $R_{1}=300\;\Omega ,R_{A}=15\;\Omega ,V_{app}=9.8\;V,v_{A}=3\;V\text{,}$$R_{3}=7\;\Omega ,\text{and}\;C_{0}=1\;\text{mF}$. (A and B) $R_{0}=70\;\Omega ,C_{\mu }=10^{-3}\;\text{mF},\text{and}\;\epsilon =10^{-4}$; (C and D) $R_{0}=70\;\Omega ,C_{\mu }=0.04\times 10^{-3}\;\text{mF},\text{and}\;\epsilon =4\times 10^{-3}$; and (E and F) $R_{0}=50\;\Omega ,C_{\mu }=10^{-3}\;\text{mF},\text{and}\;\epsilon =10^{-4}$. (G and H) Bifurcation characteristics. (G) Trace and determinant of the Jacobian, as a function of bifurcation parameter $R_{0}$ ($T_{\lambda }$ in kHz, $\Delta_{\lambda }$ in 10^5^ Hz^2^). The continuous lines are for $C_{0}=1\;\text{mF}$ and the dashed lines for $C_{0}=0.05\;\text{mF}$. The blue points indicate the Hopf bifurcations at $R_{0H1}=61.20\;\Omega$ and $R_{0H2}=75.59\;\Omega$. (H) The trace as a function of the capacitor $C_{0}$ for $R_{0}=70\;\Omega$.

In Figures 4A–4D based on Equation 23, we observe examples of the oscillatory dynamics. The nullclines are obtained by the conditions $\dot{u}=\dot{v}=0\text{.}$ For $\dot{u}=F=0$, it is

(Equation 33) $$u=\frac{R_{0}}{R_{0}+R_{1}+R_{t}}V_{app}\text{,}$$

and for $\dot{v}=G=0$,

(Equation 34) $$u=\frac{R_{1}+R_{t}}{R_{1}}v-\frac{R_{t}}{R_{1}}V_{app}\text{.}$$

The oscillation occurs when the $\dot{v}=0$ nullcline (yellow) intersects with the intermediate branch of the S-shaped $\dot{u}=0$ line (green) in a region where du/dv<0. This is an alternative expression to that of Figure 3A, in terms of the intrinsic dynamical variables. When the $R_{0}$ decreases, it moves the yellow nullcline downwards and produces a stable point in Figures 4E and 4F that corresponds to the stable intersection (green point) in Figure 3A.

The system (Equations 31 and 32) forms a typical slow-fast system determined by two relaxation times: one for the intrinsic charging of the OECT, $\tau_{\mu }=R_{3}C_{\mu }$, and another one for the charge-discharge of the external circuit, $\tau_{0}=R_{0}C_{0}$. The u is the slow stabilizing variable, and v is the fast-destabilizing variable; hence, $\tau_{0}>\tau_{\mu }$. A parameter featuring the oscillatory properties is the time ratio

(Equation 35) $$\epsilon =\frac{\tau_{\mu }}{\tau_{0}}\text{.}$$

In Figures 4A and 4B, with $\epsilon =10^{-4}$, the system develops standard relaxation oscillations, in which the system leaves and returns to a quasi-equilibrium line by fast, sudden transitions.^61^ The evolution in Figure 4A tracks the green $\dot{u}=0$ curve until a fold point is reached, and then a sudden jump occurs to the other stable branch. Since du/dt≪dv/dt, the jump consumes a negligible time, and the spikes shown in Figure 4B have a rickshaw form. In summary, relaxation oscillations are obtained provided that ϵ→0.

In Figures 4C and 4D, the $\tau_{0}$ is smaller and $\epsilon =4\times 10^{-3}$, close to the bifurcation limit $\epsilon_{B}$ established below. Now, the transition between the lower and upper branches takes significant time, and the oscillations of u(t) become close to the sinusoidal form that occurs at the Hopf bifurcation.^8^

#### Bifurcation conditions

To analyze the bifurcation properties that establish the range of oscillatory domains, we consider a linear stability analysis.^8^^,^^27^^,^^28^ The eigenvalues λ of the motion are determined by the equation

(Equation 36) $$\lambda^{2}-T_{\lambda }\lambda +\Delta_{\lambda }=0\text{,}$$

where $T_{\lambda }$ is the trace and $\Delta_{\lambda }$ is the determinant of the Jacobian of Equations 31 and 32. The Hopf bifurcation occurs when a pair of eigenvalues becomes purely imaginary, i.e., the real part of the eigenvalue changes sign from negative to positive, while the determinant is positive.

Calculating the partial derivatives of Equations 31 and 32, $F_{u}=\partial F/\partial u$, etc., we obtain, in the methods section, the matrix elements of the Jacobian. After some algebra, we find the expressions of the trace

(Equation 37) $$T_{\lambda }=F_{u}+G_{v}=-\frac{1}{C_{0}}\left[\frac{1}{R_{0}}+\frac{1}{R_{1}+R_{t}}\left(1+\frac{R_{1}}{R_{3}}\right)\right]-\frac{1}{{R_{3}C}_{\mu }}\;\left[-1+\frac{R_{1}\;R_{t}^{\prime }}{{\left(R_{1}+R_{t}\right)}^{2}}\left(V_{app}-u\right)\right]$$

and the determinant

(Equation 38) $$\Delta_{\lambda }{=F}_{u}G_{v}-F_{v}G_{u}=\frac{1}{R_{0}C_{0}{R_{3}C}_{\mu }}\;\left[1+\frac{R_{0}}{R_{1}+R_{t}}-\frac{R_{1}\;R_{t}^{\prime }}{{\left(R_{1}+R_{t}\right)}^{2}}\left(V_{app}-u\right)\right]\text{.}$$

In Figure 4G, we plot the trace and determinant as a function of the bifurcation parameter $R_{0}$. Since it is $\Delta_{\lambda }>0$, the region of $T_{\lambda }\geq 0$ produces oscillations. The bifurcation points (blue dots) in Figure 4G correspond approximately to the fold points of the $u\left(I_{0}\right)$ curve in Figure 3B. Outside this region of Figure 4G, it is $T_{\lambda }<0$, and the trajectory leads to a stable point (see Figure 3D).

However, the conditions in Figure 3B are not enough to ensure oscillation, since the system needs to have the slow/fast property mentioned before. In the dashed lines of Figure 4G, we have decreased the $C_{0}$, and the oscillatory region narrows and can eventually disappear. Figure 4H shows that the oscillations cannot happen when $C_{0}<C_{0B}$ where $T_{\lambda }\left(C_{0B}\right)=0$. The $C_{0B}$ is a lower bound to the value of the capacitor $C_{0}$ that produces oscillations, which is given below.

We conclude that the analysis of oscillatory domains based on the stationary Figure 3 is valid only for pure relaxation oscillations with $\epsilon <10^{-4}$. The methods of integration^61^^,^^62^ to find the period of relaxation oscillation, $T_{R}$, indicate that it is $T_{R}\approx \tau_{0}$, which is confirmed in Figure 4B.

### Model with realistic OECT parameters

The previous figures that elaborated on the arbitrary mobility function of Equation 20 have shown the structural properties of the bifurcation and oscillation properties. Based on these insights, we can construct the same oscillatory functionality with a realistic set of parameters describing an ordinary OECT. These are listed in Table 1.

**Table 1:** Parameters of the oscillatory OECT

Parameter	Abbreviation	Unit
Channel length	*L*	100 μm
Thickness	d	100 nm
Width	w	10 μm
Hole mobility	$\mu_{0}$	0.2 cm^2^ V s
Drain-source voltage	$V_{ds}$	0.1 V
Charge density	$a_{0}$	5 × 10^19^ cm^−3^
Chemical capacitance	$C_{\mu }$	0.8 nF
Resistance parameter	$R_{A}$	0.625 MΩ
Electrolyte resistance	$R_{3}$	1 kΩ
Series resistance	$R_{1}$	4 MΩ

In general, it is required that the mobility has a peaked shape, decreasing at high voltage, as commented before. We suggest a Gaussian mobility

(Equation 39) $$M\left(v\right)=\mathrm{Exp}\left[-\frac{{\left(v-V_{0}\right)}^{2}}{2V_{1}}\right]\text{.}$$

**Figure 5:**
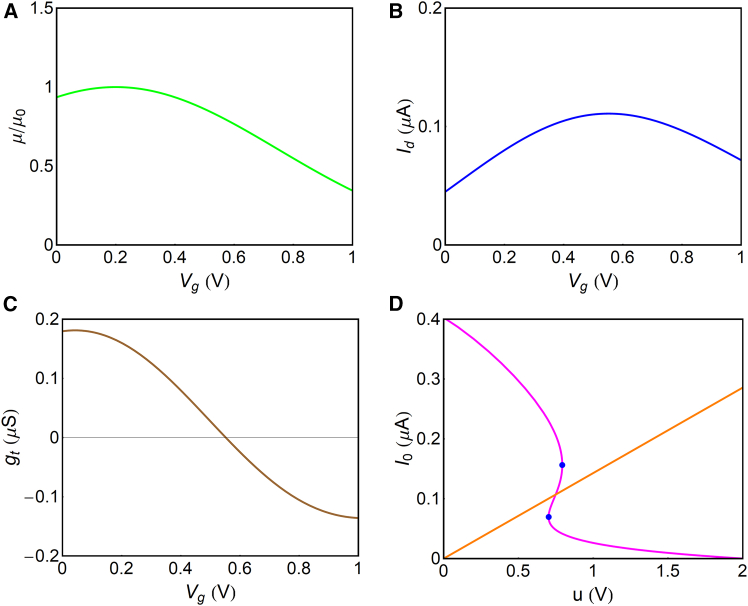
Gaussian mobility model (A–C) Mobility (A), transfer curve (B), and differential transconductance $g_{t}=dI_{ds}/dV_{g}$ (C). (D) The stationary current $I_{0}$ from $u\left(I_{0}\right)$ (magenta), and from $u/R_{0}$ (orange), with $R_{0}=7\;M\Omega$. The blue points are the folding points.

It is shown in Figure 5A. The drain current with near-Gaussian shape similar to experimental curves of antiambipolar OECT materials^10^ is shown in Figure 5B, and the negative transconductance

(Equation 40) $$g_{t}=\frac{dI_{ds}}{dV_{g}}$$

is shown in Figure 5C. By including this OECT model in the oscillatory circuit of Figure 2C, the system is described by Equations 31 and 32, and we obtain a Z-shape that can be intercepted by the load line, as shown in Figure 5D. The oscillation is possible between the fold (blue) points that signal the region of negative transconductance.

**Figure 6:**
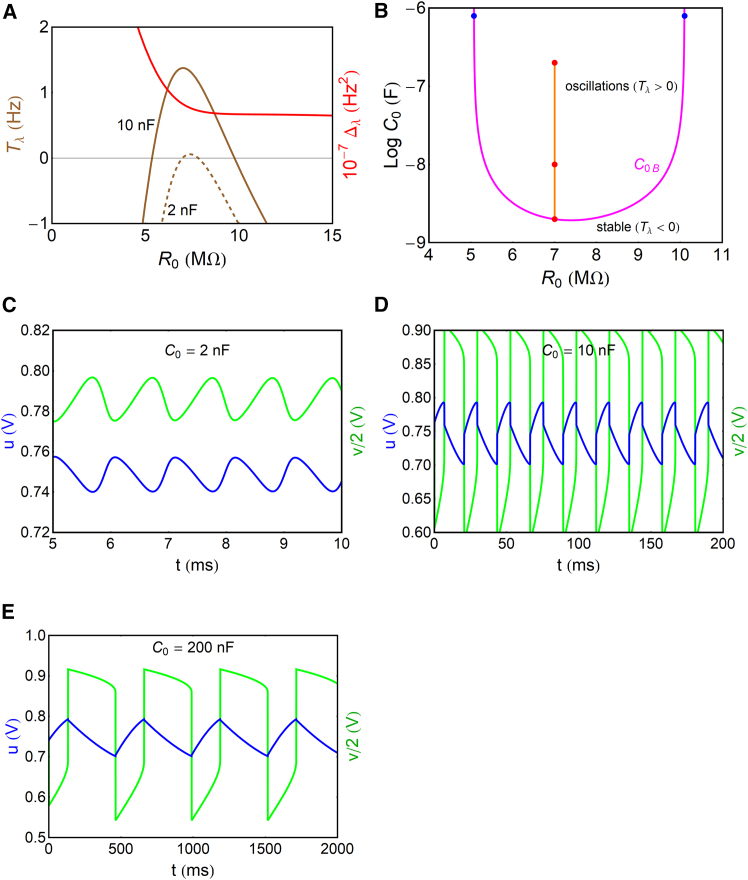
Oscillatory properties of the Gaussian model (A) Trace and determinant of the Jacobian matrix for two different values of $C_{0}$. (B) Bifurcation diagram $C_{0}\left(R_{0}\right)$. The blue points are the fold points of Figure 5C. The red points are where the oscillations of the variables u,v are shown in (C)–(E) for $R_{0}=7\;M\Omega$. Parameters are listed in Table 1.

Now, we apply the analysis of the trace and determinant given in Equations 37 and 38. The result in Figure 6A shows a region with $T_{\lambda }\geq 0,\Delta_{\lambda }>0$. This region occurs when $R_{0}$ at the stated $V_{app}$ produces the intercept of Figure 5D between the folding points, as already mentioned. However, as discussed before, a decreasing value of the capacitor $C_{0}$ narrows the bifurcation domain, as shown in the dashed line of Figure 6A. This provides another constraint: if $C_{0}$ is too small, $T_{\lambda }\geq 0$ is not possible. To quantify this condition, we can define the capacitor value $C_{0B}$ that causes the Hopf bifurcation, setting $T_{\lambda }=0$ in Equation 37; thus,

(Equation 41) $$C_{0B}=\frac{{R_{3}C}_{\mu }\left[\frac{1}{R_{0}}+\frac{1}{R_{1}+R_{t}}\left(1+\frac{R_{1}}{R_{3}}\right)\right]}{1-\frac{R_{1}\;R_{t}^{\prime }}{{\left(R_{1}+R_{t}\right)}^{2}}\left(V_{app}-u\right)}\text{.}$$

Furthermore, we have

(Equation 42) $$\epsilon_{B}=\frac{{R_{3}C}_{\mu }}{R_{0}C_{0B}}\text{.}$$

The bifurcation diagram is shown in Figure 6B. It indicates the minimum value of the capacitor that can produce oscillations, at the Hopf bifurcation, between the folding points of the resistance. The bifurcation with respect to capacitance is experimentally observed in electrochemical oscillations.^63^

Figure 6B shows the effect of changing $C_{0}$ at fixed $R_{0}$ (red points), and we calculate the corresponding oscillations in Figures 6C–6E. At the Hopf bifurcation, $C_{0}=C_{0B}$, the oscillations are nearly sinusoidal. When the capacitor increases, the system produces relaxation oscillations, where the period increases, approximately, as $T_{0}=R_{0}C_{0}$.

## Discussion

In previous work, the OECT-based neuron has been built using two organic transistors to establish the two branches of Figure 3A.^14^^,^^15^ Another method is to use the feedback by an inverting amplifier to keep the system in the negative resistance region of a Gaussian-shaped transfer curve.^10^ We have shown that sinusoidal and relaxation oscillations can be obtained with a single transistor and elementary matched elements, using the circuit of Figure 1B.

We discuss the conditions for experimental realization of the system. The main ingredient is an OECT with the peaked-shape transfer curve shown in Figure 5B. This feature introduces the essential negative transconductance that destabilizes the system. Then, we have external parameters $R_{1},R_{0},{\text{and}\;V}_{app}$ to obtain the Z-shaped curve of Figure 5D. This characteristic can be investigated by measurements in stationary conditions, removing the capacitor $C_{0}$ as in Figure 2D. As discussed before, a line from the origin must intersect $u\left(I_{0}\right)$ uniquely in the unstable branch where $du/dI_{0}>0$. Changing the operation point $V_{app}$ leads to the determination of the external voltage range and series resistance $R_{1}$ that enable this condition. Therefore, $V_{app}$ and $R_{1}$ are bifurcation parameters, in addition to $R_{0}\;\text{and}\;C_{0}$.^40^

We have shown that the dynamical conditions also require a condition of timescales of the system such that $\tau_{\mu }<0.01\;\tau_{0}$ (approximately). This restriction can be obtained by modifying the parameters $R_{3},C_{\mu },\text{and}\;C_{0}$. For the $\tau_{\mu }$ to be small, both the internal resistance $R_{3}$ and the volume capacitance $C_{\mu }$ of the OECT must be small, so the channel needs to be thin. On the other hand, the $C_{0}$ can be set at will, which enables tuning the frequency of the spikes. The oscillations disappear when the external capacitor becomes small, limiting the possible oscillation frequencies. The construction of Figure 1B can be extended to other technologies that show negative transconductance, such as TFT.^59^

In practice, the transfer curve characteristics and the dynamical response of OECT can be more complicated than the simple exploratory $R_{3}C_{\mu }$ model of Figure 2B that we have used here, as we have shown in recent work.^32^^,^^33^ In fact, the organic transistor can contain different internal capacitors and resistors.^64^^,^^65^ These internal dynamical properties, when combined with the model of Figure 2, may lead to richer properties of spiking that are useful in a biological context, like different types of bursting.^66^ This subject needs further investigation.

To advance organic electrochemical neuromorphic circuits for real-world use, designs must stay functional while reducing complexity. Our one-transistor RC-based approach simplifies circuit design. Key benefits include (1) low-cost fabrication via flexible, low-temperature processes like printing; (2) energy efficiency from minimal component use and event-driven operation; (3) biomimicry—organic transistors emulate neural behaviors through ionic-electronic coupling and nonlinear dynamics; (4) ultra-compact size, reducing the neuron area compared to CMOS designs; and (5) mechanical flexibility, ideal for wearable or implantable systems. Negative factors include (1) limited gain and signal strength, as organic transistors have low transconductance and current drive without amplifiers; (2) high variability and low stability due to sensitivity to moisture and oxygen; (3) slower switching speeds from low carrier mobility, limiting high-speed use—though this suits low-frequency biological signals; and (4) limited integration density, as large networks may face signal integrity issues without buffers or amplifiers.

A variety of approaches produce biocompatible spiking neurons based on OECTs. Here, we leverage the intrinsic mobility dependence of many organic ionic-electronic conductors to produce a circuit that provides self-sustained oscillations using just one organic transistor and complementary RC external elements. The transistor delivers the fast variable and the external RC the slow stabilizing element of the dynamics, while a series resistor modulates the effect of the external potential. Using linear response theory in an elementary transistor model formed by a reduced set of differential equations, it is possible to fully characterize the Hopf bifurcation properties in terms of the physical parameters. We express experimental criteria to obtain the oscillations, and we suggest that transistors with more complicated internal dynamic features can give rise to rich spiking and bursting patterns. Using a single organic transistor for a neuron device—without supporting amplifiers—is promising for energy-efficient, biomimetic, and low-cost neuromorphic systems, especially in low-power, biocompatible, and flexible electronics.

## Methods

### S-type oscillator dynamical equations

In the case of Figures 1A and 1C, the fast variable is the measured voltage u, with a dynamic control by the external capacitor, and the slow variable x is an internal state variable of the memristor that governs the bistable behavior. A model, recently reviewed,^8^ can be stated as

(Equation 43) $$C_{0}\frac{du}{dt}=I_{0}-g\left(w\right)u$$

(Equation 44) $$\tau_{k}\frac{dw}{dt}=g\left(w\right)u-w\text{.}$$

Here, the slow variable is $w=I_{1}=g\left(w\right)u$, where g(w) is the conductance of the nonlinear element, and $\tau_{k}$ is the relaxation time. The main condition for oscillations is that the load line of the circuit of Figure 1A,

(Equation 45) $$I_{0}=\frac{V_{app}-u}{R_{1}}\text{,}$$

intersects the S-shape in a negative resistance point.

In the model of Equations 43 and 44, there is no tank circuit in the mechanism of a chemical inductor, as the inductor element is provided by the nonlinear device.^67^^,^^68^ Only the external capacitor is needed to produce a limit-cycle oscillator.

### Derivation of the dynamical equations

The first equation is

(Equation 46) $$C_{\mu }\frac{dv}{dt}=\frac{1}{R_{3}}\left(V_{g}-v\right)\text{.}$$

From the main text,

(Equation 47) $$V_{g}=V_{app}-R_{1}I_{0}\text{,}$$

(Equation 48) $$I_{0}=\frac{V_{g}-u}{R_{t}}\text{,}$$

(Equation 49) $$V_{app}=u+\left(R_{1}+R_{t}\right)I_{0}$$

(Equation 50) $$I_{0}=\frac{V_{app}-u}{R_{1}+R_{t}}\text{.}$$

Thus,

(Equation 51) $$\frac{dv}{dt}=\frac{1}{R_{3}C_{\mu }}\left(V_{app}-R_{1}I_{0}-v\right)\text{,}$$

with the final expression

(Equation 52) $$\frac{dv}{dt}=\frac{1}{R_{3}C_{\mu }}\left(-v+\frac{R_{1}}{R_{1}+R_{t}}u+\frac{R_{t}}{R_{1}+R_{t}}V_{app}\right)\text{.}$$

The second equation,

(Equation 53) $$I_{0}=I_{1}+I_{2}\text{,}$$

can be written as

(Equation 54) $$\frac{V_{app}-u}{R_{1}+R_{t}}=C_{0}\frac{du}{dt}+\frac{u}{R_{0}}+C_{\mu }\frac{dv}{dt}\text{.}$$

Hence,

(Equation 55) $$C_{0}\frac{du}{dt}=-\frac{u}{R_{0}}+\frac{V_{app}-u}{R_{1}+R_{t}}-C_{\mu }\frac{dv}{dt}\text{.}$$

### Components of the Jacobian matrix

(Equation 56) $$J=\left[\begin{array}{cc} F_{u} & F_{v}\\ G_{u} & G_{v} \end{array}\right]$$

(Equation 57) $$F_{u}=\frac{\partial F}{\partial u}=-\frac{1}{C_{0}}\left(\frac{1}{R_{0}}+\frac{1}{R_{1}+R_{t}}\right)-\frac{C_{\mu }}{C_{0}}G_{u}$$

(Equation 58) $$F_{v}=\frac{\partial F}{\partial v}=-\frac{1}{C_{0}}\;\frac{R_{t}^{\prime }}{{\left(R_{1}+R_{t}\right)}^{2}}\left(V_{app}-u\right)-\frac{C_{\mu }}{C_{0}}G_{v}$$

(Equation 59) $$G_{u}=\frac{\partial G}{\partial u}=\frac{1}{{R_{3}C}_{\mu }}\frac{R_{1}}{R_{1}+R_{t}}$$

(Equation 60) $$G_{v}=\frac{\partial G}{\partial v}=\frac{1}{{R_{3}C}_{\mu }}\;\left[-1+\frac{R_{1}\;R_{t}^{\prime }}{{\left(R_{1}+R_{t}\right)}^{2}}\left(V_{app}-u\right)\right]$$

## Resource availability

### Lead contact

Further information and requests for resources and reagents should be directed to and will be fulfilled by the lead contact, Juan Bisquert (jbisquer@itq.upv.es).

### Materials availability

This study did not generate new unique materials.

### Data and code availability

The data presented here can be accessed at Zenodo (https://doi.org/10.5281/zenodo.15244569) under the license CC BY 4.0 (Creative Commons Attribution-ShareAlike 4.0 International).

## Acknowledgments

This work was funded by the 10.13039/501100000781European Research Council (ERC) via a Horizon Europe Advanced Grant, grant agreement no. 101097688 ("PeroSpiker").

## Author contributions

J.B. conceived and supervised this project and performed the primary calculations and simulations. N.T. performed analyses of the oscillator model. J.B. and N.T. wrote the manuscript. Both authors contributed to the final version of the manuscript.

## Declaration of interests

The authors declare no competing interests.

## Declaration of generative AI and AI-assisted technologies in the writing process

During the preparation of this work, the authors used ChatGPT 4o in order to improve the introduction and conclusions. After using this tool/service, the authors reviewed and edited the content as needed and take full responsibility for the content of the publication.

## Supplemental information

Document S1. Figures S1–S3

Document S2. Article plus supplemental information
