# Supplementary material for: A one-transistor organic electrochemical self-sustained oscillator model for neuromorphic networks
Source: Newton. 2025 Oct 6;1(8):None. doi: 10.1016/j.newton.2025.100207 (PMC12501206; doi:10.1016/j.newton.2025.100207)
Supplement: Document S1. Figures S1–S3 [file mmc1.pdf]

NEWTON, Volume *1*

Supplemental information

A one-transistor organic electrochemical self-sustained oscillator model for neuromorphic networks

Juan Bisquert and Nir Tessler

Supplementary Methods

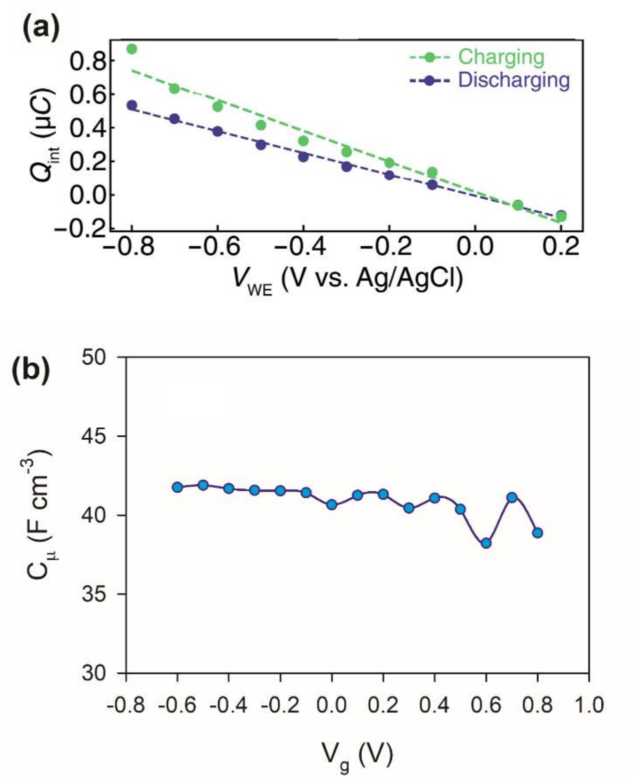

Fig. S1. **Charging OMIEC films of PEDOT:PSS.** (a) Integrated charge extracted during charging and injected during discharging for experiments at varied potentials showing a linear dependence between charge and the applied potential. Reproduced from Keene, S. T.; Rao, A.; Malliaras, G. G. The relationship between ionic-electronic coupling and transport in organic mixed conductors, Science Advances 2023, 9, eadi3536, Fig. 2. Licensed under CC BY 4.0.[1] (b) Chemical (volume capacitance measured with AC impedance method. Reproduced from Bisquert, J.; Keene, S. T., *Advanced Science* **2025**, *12*, 2410393, licensed under a Creative Commons Attribution (CC BY 4.0) license.[2]

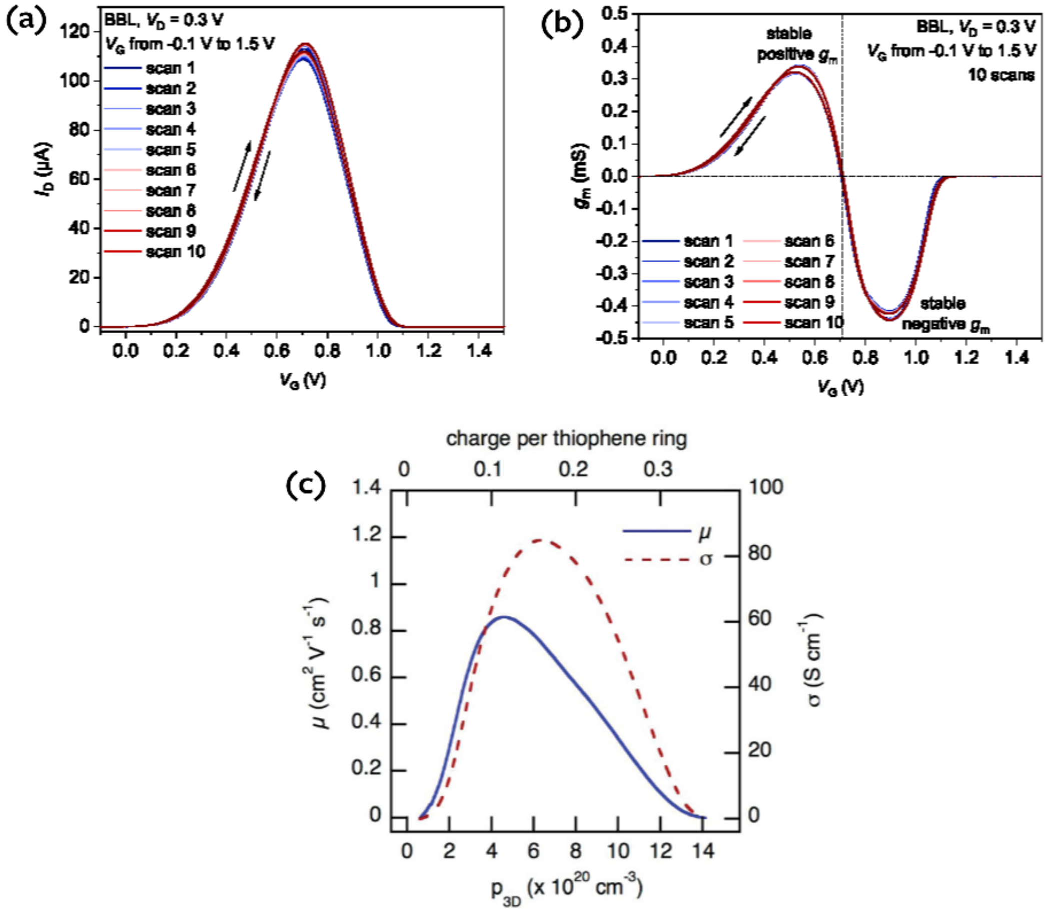

Fig. S2. **Negative differential conductance and transconductance in organic films.** (a) Stable drain current and (b) negative transconductance in BBL (Poly(benzimidazobenzophenanthroline)). Reproduced from Harikesh, P. C.; Yang, C.-Y.; Wu, H.-Y.; Zhang, S.; Donahue, M. J.; Caravaca, A. S.; Huang, J.-D.; Olofsson, P. S.; Berggren, M.; Tu, D.; Fabiano, S. Ion-tunable antiambipolarity in mixed ion–electron conducting polymers enables biorealistic organic electrochemical neurons, *Nat. Mater.* **2023**, *22*, 242–248, licensed under a Creative Commons Attribution (CC BY 4.0) license.[3] (c) Charge density dependent hole mobility and conductivity of a P3HT film.

Reprinted with permission from Bryan D. Paulsen and C. Daniel Frisbie, The Journal of Physical Chemistry C, 2012, 116, 3132–3141 (Fig. 4). Copyright 2012 American Chemical Society.[4]

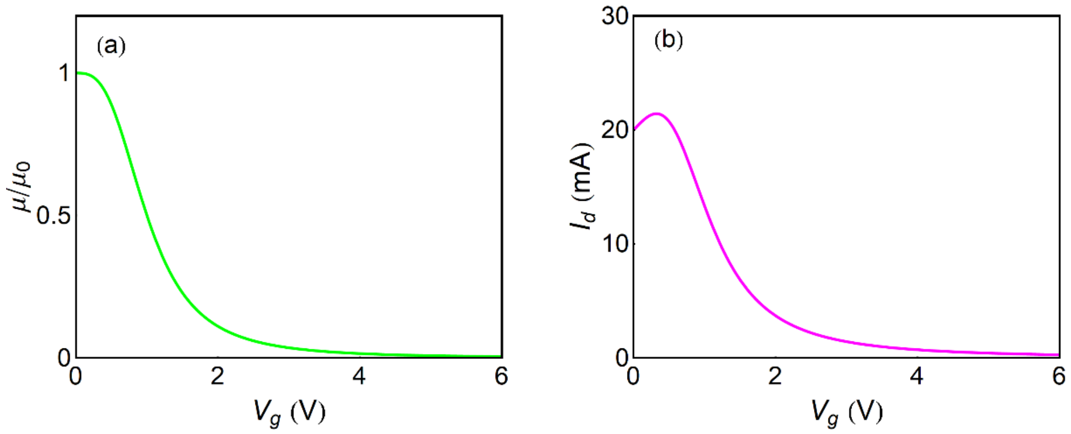

Fig. S3. **Negative differential mobility and transconductance in the model**. (a) Mobility and (b) transfer curve for $\mu(v) = \mu_0/(1 + v^3)$. Parameter values: $\mu_0 = 1\, m^2/Vs,\ \ R_A = 15\ \Omega V, v_A = 3\ V,\ V_{ds} = -1\ V$.

Supplementary References

(1) Keene, S. T.; Rao, A.; Malliaras, G. G. (2023) The relationship between ionic-electronic coupling and transport in organic mixed conductors, *Science Advances* , *9*, eadi3536.

(2) Bisquert, J.; Keene, S. T. (2025) Using the Transversal Admittance to Understand Organic Electrochemical Transistors, *Advanced Science*, *12*, 2410393

(3) Harikesh, P. C.; Yang, C.-Y.; Wu, H.-Y.; Zhang, S.; Donahue, M. J.; Caravaca, A. S.; Huang, J.-D.; Olofsson, P. S.; Berggren, M.; Tu, D.; Fabiano, S. (2023) Ion-tunable antiambipolarity in mixed ion–electron conducting polymers enables biorealistic organic electrochemical neurons, *Nat. Mater.*, *22*, 242–248.

(4) Paulsen, B. D.; Frisbie, C. D. (2012) Dependence of Conductivity on Charge Density and Electrochemical Potential in Polymer Semiconductors Gated with Ionic Liquids, *J. Phys. Chem. C*, *116*, 3132-3141.
